# Transcriptional changes and the role of ONECUT1 in hPSC pancreatic differentiation

**DOI:** 10.1038/s42003-021-02818-3

**Published:** 2021-11-17

**Authors:** Sandra Heller, Zhijian Li, Qiong Lin, Ryan Geusz, Markus Breunig, Meike Hohwieler, Xi Zhang, Gopika G. Nair, Thomas Seufferlein, Matthias Hebrok, Maike Sander, Cécile Julier, Alexander Kleger, Ivan G. Costa

**Affiliations:** 1 Department of Internal Medicine I, Ulm University Hospital, Ulm, Germany; 2 Institute for Computational Genomics, RWTH Aachen University Medical School, Aachen, Germany; 3 Bayer AG, Research & Development, Pharmaceuticals, Bioinformatics, Berlin, Germany; 4 Pediatric Diabetes Research Center (PDRC) at the University of California, San Diego, USA; 5 Diabetes Center at the University of California, San Francisco, USA; 6 Université de Paris, Institut Cochin, INSERM U1016, CNRS UMR-8104, Paris, France

**Keywords:** High-throughput screening, Stem-cell differentiation, Differentiation

## Abstract

Cell type specification during pancreatic development is tightly controlled by a transcriptional and epigenetic network. The precise role of most transcription factors, however, has been only described in mice. To convey such concepts to human pancreatic development, alternative model systems such as pancreatic in vitro differentiation of human pluripotent stem cells can be employed. Here, we analyzed stage-specific RNA-, ChIP-, and ATAC-sequencing data to dissect transcriptional and regulatory mechanisms during pancreatic development. Transcriptome and open chromatin maps of pancreatic differentiation from human pluripotent stem cells provide a stage-specific pattern of known pancreatic transcription factors and indicate ONECUT1 as a crucial fate regulator in pancreas progenitors. Moreover, our data suggest that ONECUT1 is also involved in preparing pancreatic progenitors for later endocrine specification. The dissection of the transcriptional and regulatory circuitry revealed an important role for ONECUT1 within such network and will serve as resource to study human development and disease.

## Abstract (web summary)

Heller et al analyze transcriptomic and epigenetic datasets from human PSCs differentiating into pancreatic progenitors to elucidate the regulatory mechanisms behind pancreatic development. The authors report that the transcription factor ONECUT1 is expressed specifically in pancreatic development and associates with other key TFs (such as FOXA2, GATA6, PDX1) during endocrine specification.

## Introduction

Damage to the endocrine pancreas can cause several forms of diabetes mellitus. Diabetes, as one of the major diseases in industrial countries, affects over 350 million people worldwide. Type 1 (T1D) and type 2 diabetes (T2D) are the most common forms and have a multifactorial etiology, but the current classification does not cover the large clinical and biological variability of the disease^1,2^. While T1D is a chronic autoimmune disease affecting insulin-producing β-cells, T2D is a metabolic disease. In T2D, insulin deficiency is caused by insulin resistance in target organs and pancreatic β-cell failure. The onset and pathophysiology of diabetes is influenced by a complex interplay of several factors such as environment, genetic predisposition, and immune system. Additionally, a small subset of diabetes patients, generally estimated to account for approximately 1–5% of cases, is caused by monogenic mutations^3,4^ altering the development, function or survival of β-cells through a variety of mechanisms. Interestingly, frequent variants of several genes, whose mutations cause monogenic diabetes, are also associated with an increased risk of multifactorial diabetes. Therefore, the characterization of pancreatic differentiation and the study of genes and regulatory pathways involved in pancreatic endocrine development and function are extremely valuable to discover potential candidates contributing to the development of different forms of diabetes.

Binding of transcription factors (TFs) at specific promoters, enhancers, and repressors controls gene expression and thus, drives cellular differentiation. Understanding the transcriptional regulations that underlie cell fate decisions requires the characterization of TF binding sites across multiple differentiation stages. Pancreatic in vitro differentiation of human pluripotent stem cells (PSC) constitutes a suitable human model system with access to distinct developmental stages^5–12^. In turn, different forms of diabetes have been appropriately modeled by PSC differentiations^13^. Sequencing techniques such as RNA and Assay for Transposase Accessible Chromatin sequencing (RNA-seq and ATAC-seq) can be applied at distinct differentiation stages to allow the global characterization of transcriptional and regulatory changes during pancreatic differentiation. Moreover, computational analysis of ATAC-seq data such as digital footprinting^14,15^ can be applied to systematically identify cis-regulatory regions and putative TF binding sites controlling stage-specific regulation. With chromatin immunoprecipitation DNA sequencing (ChIP-seq) the actual binding of specific TFs to such regulatory gene clusters can be determined.

Here, we applied stage-specific RNA-, ChIP-seq, and ATAC-seq experiments during PSC-based differentiations to dissect transcriptional and regulatory mechanisms during pancreatic development. We specifically focused on the functional role of the TF ONECUT1 and found an important role as regulator of pancreas progenitor differentiation in our analysis. In our accompanying study, we could additionally demonstrate that mutations in ONECUT1 contribute to a broad spectrum of diabetes^16^.

## Results

### Transcriptome maps of pancreas differentiation from human pluripotent stem cells

**Fig. 1:**
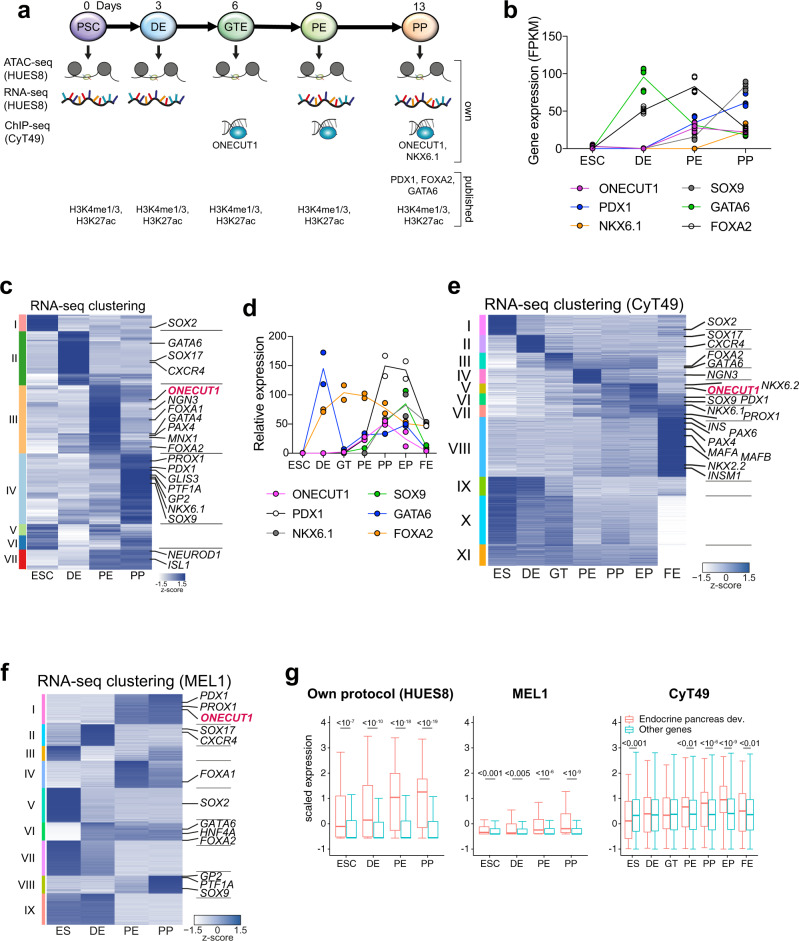
Stage-specific transcriptome analysis reveals *ONECUT1* expression during stem cell-based pancreatic differentiation. **a** Schematic outline of applied pancreatic differentiation strategy of human pluripotent stem cells and subsequent stage-specific large-scale sequencing analysis. RNA-seq data (own and E-MTAB-1086), ATAC-seq data (own), ChIP-seq data of ONECUT1 (own), FOXA1, FOXA2, PDX1 (GSE54471, GSE149148) and NKX6.1 (own), and histone data (H3K4me1, H3K27ac, GSE54471; H3K4me3, ArrayExpress E-MTAB-1086) were used for analysis. **b** Stage-specific expression analysis of depicted genes during pancreas differentiation of HUES8 cells (RNA-seq, *n* = 6 biologically independent samples). **c** Fuzzy c-means (FCM) clustering of the differentially expressed genes (RNA-seq) at the varying differentiation stages of hESCs (HUES8) displaying selected genes at respective clusters. **d** Stage-specific expression analysis of depicted genes during pancreas differentiation of CyT49 cells^30^ (RNA-seq, *n* = 2 biologically independent samples). **e** Heatmap of fuzzy c-means clustering of differentially expressed genes (RNA-seq) at distinct pancreatic differentiation stages of CyT49 cells^30^. Selected genes of particular clusters are highlighted. **f** Heatmap of fuzzy c-means clustering of differentially expressed genes (RNA-seq, *n* = 4) at the distinct pancreas differentiation stages of MEL1. Selected genes of particular clusters are displayed. Of note, cells at PP stage were purified for PDX1 and NKX6.1. **g** Expression of genes associated with endocrine pancreas differentiation obtained from Gene Ontology (ID GO:0031018) for all protocols. Boxplots depict median (center), interquartile range (box) and extreme values (whiskers). A statistical test (Wilcoxon Rank Sum test; one sided) was used to compare the expression of endocrine genes vs. all other genes.

Notably, human pancreatic development is faithfully recapitulated by differentiation of PSCs into the pancreatic lineage^5–7,11,17^. We acquired stage-specific RNA-seq, ATAC-seq, and ChIP-seq data in such a human stem cell-based differentiation approach to characterize pancreatic development (Fig. 1a). Stage-specific modulation of signaling pathways such as Wnt and Hedgehog differentiates PSCs from embryonic stem cell stage (ESC) towards definitive endoderm (DE), followed by gut tube endoderm (GT), pancreatic endoderm (PE), and pancreatic progenitors (PP). Key TFs and cellular markers were indeed stage-specifically expressed (Fig. 1b, c and Supplementary Fig. 1a, b). *SOX2* (cluster I), an essential factor for pluripotency and self-renewal^18^ was expressed in pluripotent stem cells and became downregulated during DE differentiation, while in exchange markers of DE stage such as *GATA6*, *CXCR4*, and *SOX17*^19–21^ became upregulated (cluster II). Expression of *FOXA1/2*, important for pancreatic specification by regulating *PDX1* expression^22,23^, was induced during DE stage (cluster III) and its expression was sustained in PE stage. In turn, *PDX1* was upregulated at PE stage (cluster IV). Similarly, *GATA4* was expressed at PE stage and *NKX6.1*, *GP2*, *PROX1*, *PTF1A*, and *SOX9* at PP stage (cluster III, IV)^24–26^. Moreover, TFs important for later stages of endocrine development such as *GLIS3*, *NEUROD1* and *ISL1* (cluster IV, VII) started to be expressed at PE and PP stage^27,28^. To support these gene expression dynamics during pancreatic differentiation, we employed two additional human embryonic stem cell lines (Fig. 1d–f and Supplementary Fig. 1c, d). We observed a similar expression pattern of stage-specific TFs in two further data sets employing distinct cell lines^5,17,29,30^, where key markers for PSC (*SOX2*), DE (*SOX17*, *CXCR4*), PE (*PDX1*), and PP stage (*NKX6.1*) as well as other TFs such as *FOXA2*, *SOX9*, and *PROX1* were expressed at the respective stages. To evaluate the capacity of the protocols and to quantify stages closer to pancreatic endocrine cells, we performed a gene set-based analysis with developmental genes of the endocrine pancreas. The expression of endocrine genes was enriched in PE and PP stage for HUES8^9,11,12^ and MEL1 protocols^17^ and for PP and endocrine progenitor (EP) stage in the CyT49 protocol^29^ (Fig. 1g). Expression of endocrine genes was again decreased in CyT49 differentiation stages resembling fetal β-cells (FE stage). Moreover, the HUES8 protocol explored herein had the highest increase of pancreatic endocrine gene expression among all three protocols.

### Open chromatin maps of pancreas differentiation from human pluripotent stem cells

**Fig. 2:**
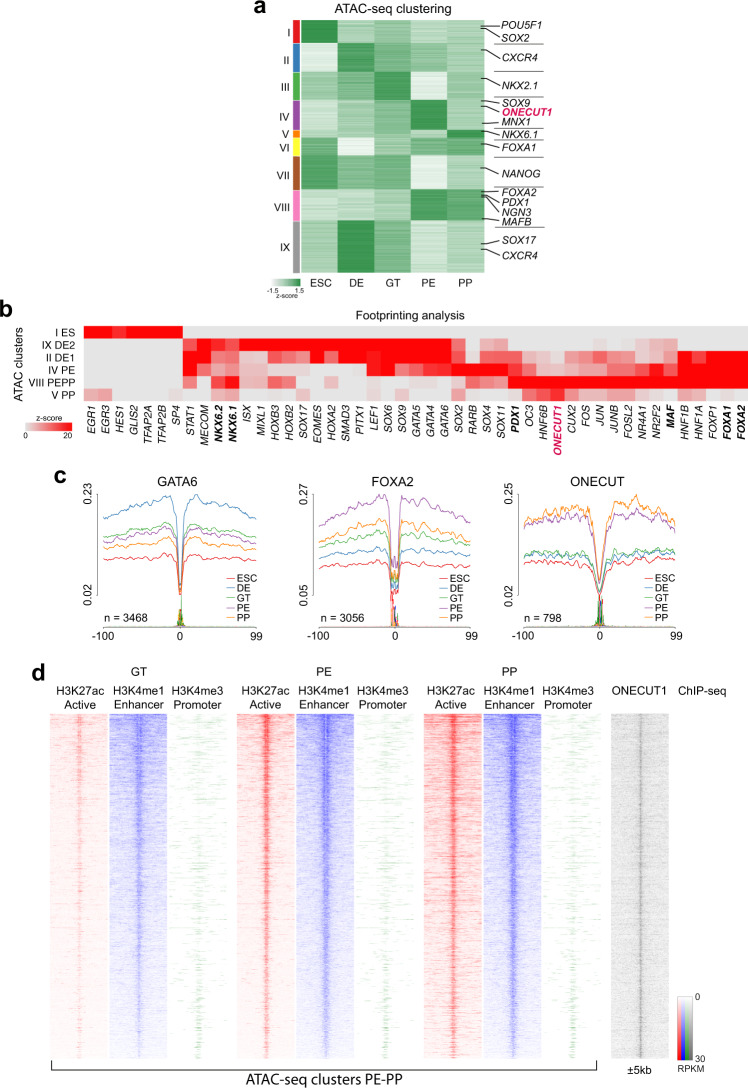
ONECUT1 binding and chromatin opening is relevant for pancreas development. **a** Fuzzy c-means clustering of the differential open chromatin peaks (ATAC-seq) during pancreatic differentiation of HUES8 cells. **b** TF binding motif enrichment at distinct open chromatin clusters of ATAC-seq. **c** Average chromatin accessibility profiles around footprint-supported bindings sites of selected TFs. **d** Heatmap ChIP-seq signals (+/− 5 kb of peak center) of H3K4me1, H3K4me3, and H3K27ac at ATAC-seq peaks from cluster PE-PP (VIII; clustering is shown in Fig. 2a). Peaks are ordered by the decrease in the H3K4me1 mark at PE stage. We also depict ONECUT1 ChIP-seq signals (PP stage) in the vicinity of the peak (+/− 5 kb).

To spatiotemporally map genome-wide chromatin accessibility during pancreatic differentiation, we performed the ATAC with massively parallel sequencing at different stages of pancreatic differentiation (Fig. 2a). First, clustering of the developmentally regulated open chromatin (OC) regions revealed stage-specific OC clusters like clusters obtained via RNA-seq (Figs. 1c and 2a). The *SOX2* locus (cluster I) was accessible only in ESC stage, whereas the loci for *CXCR4* and *SOX17* (cluster IX) became accessible during differentiation to DE stage. The *FOXA1/2* locus had limited accessibility at DE stage but opened during further development (cluster VI, VIII) like RNA expression patterns. The *NKX6.1* locus was allocated to a cluster peaking at the PP stage (Cluster V; Fig. 2a). Similarly, chromatin accessibility for other core endocrine gene loci such as *GLIS3* or *MAFB* was initiated from PE to PP stage (Cluster VIII; Fig. 2a).

Next, we investigated if TF binding motifs were stage-specifically enriched in OC loci to dissect regulatory changes between distinct TFs (Fig. 2b). This unbiased footprinting analysis indicated the activity of well-known TFs to control pancreas expression programs: GATA6 and SOX9 in early stages and NKX6.1, NKX6.2, FOXA1, FOXA2, MAF, PDX1, and ONECUT1 in later stages. This cell-specific enrichment, i.e., importance of GATA6 in early DE stage, FOXA2 in PE stage and ONECUT1 in both PE and PP stages, was illustrated by the accessibility profiles around footprinting-supported motifs (Fig. 2c). This showed an increasing activity of ONECUT1 motif with a peak on both PE and PP stages, while FOXA2 had a peak activity in PE stage only.

### ONECUT1 during human pancreas development

Following our genetic study identifying *ONECUT1* as diabetes gene involved in a broad spectrum of diabetes in human (accompanying article)^16^, we focused our further analysis on *ONECUT1*. This TF has so far been mainly characterized in mouse models^31–34^ and little is known about its role during human pancreatic differentiation^35^. *ONECUT1* expression increased from the DE stage to the PE stage with sustained expression at the PP stage (Fig. 1b, f). *ONECUT1* was assigned alongside key TFs necessary for PE development including *FOXA1/2, GATA4*, as well as *NGN3* and *MNX1* known to be important for the initiation of the β-cell program (Cluster III; Fig. 1c). Robust expression from the PE to the PP stage was reproduced on published data sets in several genetic backgrounds (MEL1^5,17^ and CyT49^29,30^ hESCs) (Fig. 1g–j). Notably, *ONECUT1* was only marginally expressed at the gut tube stage (Fig. 1f). Moreover, the *ONECUT1* locus opened around the GT and peaked at the PE stage (Fig. 2a), closely correlating with gene transcription levels (Figs. 1b, c). Additionally, motif enrichment and footprint analysis^15^ confirmed the dynamic and increasing activity of ONECUT1 from the PE to the PP stage (Fig. 2b, c**)**. While ONECUT1-bound elements within the PE/PP cluster had high levels of active enhancer marks (both H3K4me1 and H3K27ac) beginning at the PE stage, they displayed very low levels of the promoter mark H3K4me3 at all stages (Fig. 2d). Taken together, these data suggest that accessible chromatin dynamics during pancreatic cell fate acquisition follow precise stage-specific patterns.

### ONECUT1 is a crucial regulator of pancreas progenitors

**Fig. 3:**
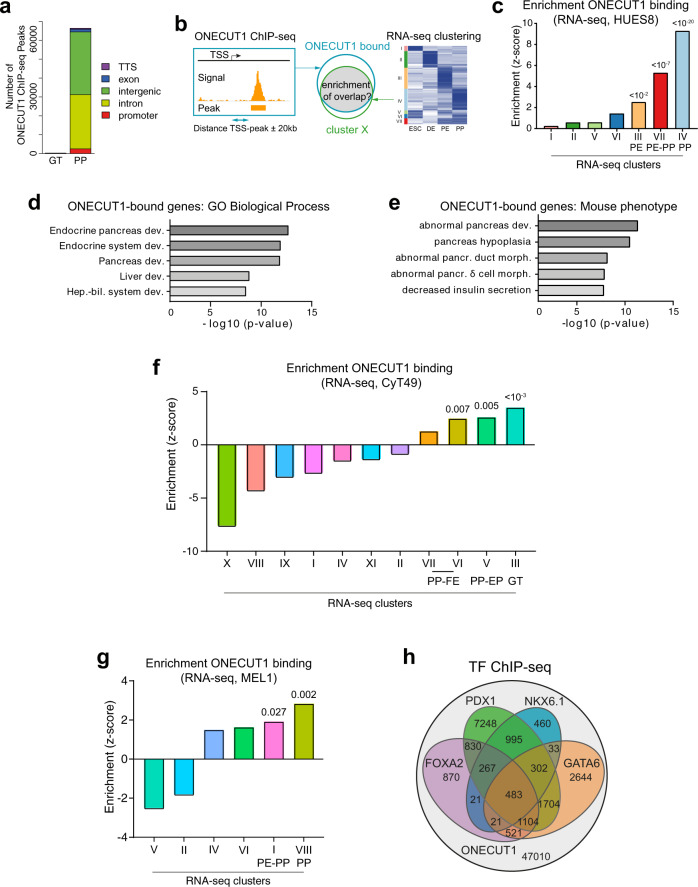
ONECUT1 binds to regulatory gene clusters relevant for pancreas and endocrine development. **a** Distribution of ONECUT1 ChIP-seq peaks at GT and PP stages relative to genomic regions. **b** Schematic of the binding enrichment analysis evaluating whether ONECUT1-bound genes (binding observed 20 kb upstream or downstream of the respective promoter regions) are enriched in particular RNA-seq clusters. **c** Binding enrichment (*z*-score) test of ONECUT1 (ChIP-seq, PP stage) in genes of the RNA-seq clusters of HUES8 cells (*z*-test; one-sided). **d**, **e** Enrichment analysis (GREAT^36^) of ONECUT1-bound genes (ChIP-seq, PP stage) (Fischer Exact test; one-sided). **f** Binding enrichment (*z*-score) test of ONECUT1 (ChIP-seq, PP stage) in genes of the RNA-seq clusters of CyT49 cells (*z*-test; one-sided). Significance is indicated above bars (Fischer Exact test; one-sided). **g** Binding enrichment test of ONECUT1 (ChIP-seq, PP) for MEL1 RNA-seq specific gene clusters. Data show binding score (*z*-score) with *p*-value (*z*-test; one-sided). **h** Overlap of ONECUT1 ChIP-seq peaks (PP stage, own data set) with peaks from other TFs (previously published at GSE54471, GSE149148, or NKX6.1; own data set). For the other TFs, only the number of binding peaks overlapping with ONECUT1 is shown.

We have recently investigated the occupancy of ONECUT1 binding at the GT and PP stages by performing ChIP-seq experiments^35^. In line with the *ONECUT1* expression profile, we observed only a few hundred ONECUT1 ChIP-seq peaks at the GT stage, predominantly at promoter sites. In contrast, at the PP stage tens of thousands of ONECUT1 ChIP-seq peaks were found, predominantly located in distal gene regions (Fig. 3a). ONECUT1-bound genes were enriched within the PE, PE-PP transition, and most abundantly in the PP gene cluster, indicating an increasingly important role of ONECUT1 during PP differentiation (Fig. 3b, c). Similar enrichment of ONECUT1 in PE and PP clusters was also observed in cells from other genetic backgrounds (Fig. 3f, g). GREAT analysis (Genomic Regions Enrichment of Annotations Tool^36^) of ONECUT1-bound genes was associated with processes such as (endocrine) pancreas and endocrine system development, decreased insulin secretion, and pancreatic hypoplasia (Fig. 3d, e).

During pancreatic cell differentiation, the state of enhancers is accompanied by a defined sequence of chromatin changes: chromatin decompaction via pioneer factors or cooperative TF binding, poising/priming via accumulation of H3K4me1 marks, and activation by deposition of H3K27ac marks. This leads to the acquisition of a cell fate-specific gene expression program^29,37–42^. To further characterize the nature of distal ONECUT1 binding sites at the PP stage (Fig. 3a), we cross-referenced our data with public and own ChIP-seq data for enhancer histone marks (active: H3K4me1/H3K27ac; poised: H3K4me1) and TFs (NKX6.1, FOXA2, GATA6 and PDX1) at the PP stage^29,30^. To characterize chromatin states associated with ONECUT1 and these TFs, we made use of a chromHMM annotation data set^43^ employing the same pancreas differentiation stages^35^. This annotation allowed us to gather additional information of active and inactive enhancers, active promoters and repressive regions among others.

**Fig. 4:**
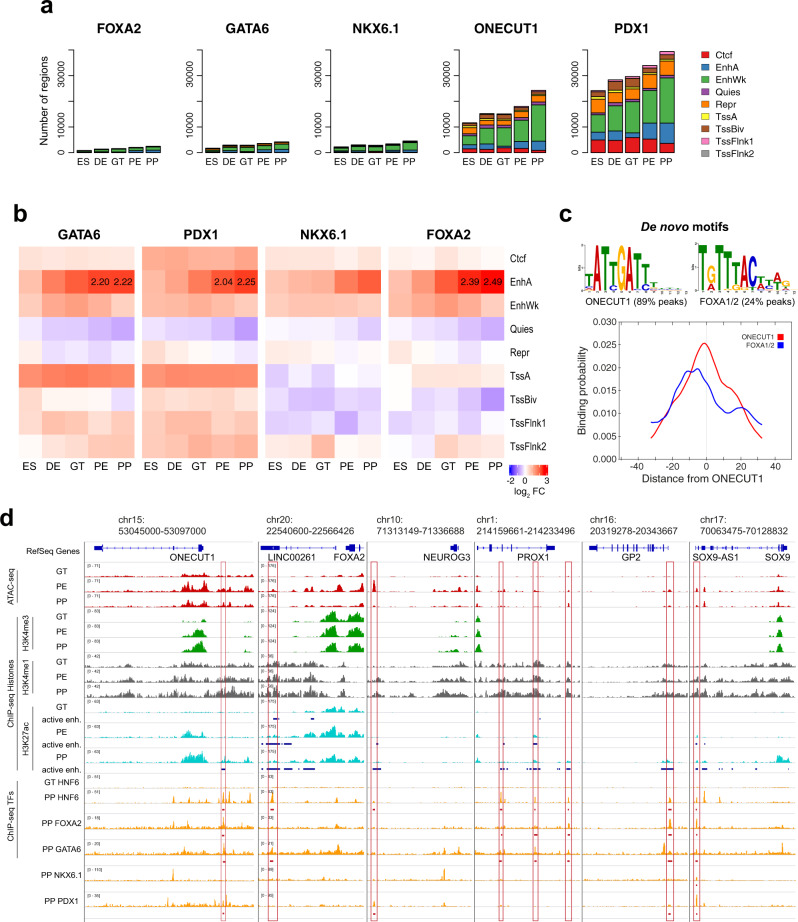
ONECUT1 cobinding during early pancreas differentiation regulation. **a** Overlap of peaks of distinct TFs on chromatin states as delineated by chromHMM. These states include active enhancers (EnhA), weak/poised enhancers (EnhWk.), repressed regions (Repr.), active promoters (TssA), poised promoters (TssBiv) and regions flanking active promoters (TssFlnk1-2). **b** Log2 fold change (FC) of the frequency of ONECUT1 co-binding events (ONECUT1 with either GATA6, PDX1, NKX6.1, and FOXA2) vs. ONECUT1 exclusive binding sites for distinct chromatin states and differentiation stages. Red values indicate chromatin stages over-represented in co-binding events. We highlight values with abs(log2(FC)) > 2. **c** We show MEME de novo motifs around ONECUT1 ChIP-seq peaks, which are found in 89% of peaks, and FOXA2 motif, which is found in 24% of peaks. Both motifs have a preference relative to the peak center, i.e., ONECUT1 is mostly localized in the peak middle, while FOXA2 is located 9–10 bps downstream of peak center. **d** IGV Genome browser view of ONECUT1 and PE/PP expressed genes with ChIP-seq and ATAC-seq profiles from GT, PE, and PP stages. We highlight ONECUT1 peaks overlapping with active enhancers (blue tracks below H3K27ac tracks) gained at PE or PP stage, as well as co-binding partners.

First, we observed an overlap of ONECUT1 peaks with PDX1, GATA6, FOXA2, and NKX6.1 in 21, 10, 6, and 4% of peaks (Fig. 3h). The evaluation of chromatin states indicated that ONECUT1 primarily bound to enhancers (strong or weak) and the number of enhancers particularly increased after the GT stage. ONECUT1 was bound to 3.6% of active enhancers existing in GT stage, 9.7% of active enhancers in PE stage and 14% of active enhancers in PP stage (Fig. 4a). Among TFs analyzed here, only PDX1 was bound to a larger number of active enhancers, e.g., 9%, 21%, and 31% for GT, PE, and PP stages, respectively (Fig. 4a). Next, we asked if co-binding of GATA6, PDX1, NKX6.1, and FOXA2 with ONECUT1 is enriched for particular chromatin states. Interestingly, we observed that co-binding events of ONECUT1 with these TFs were particularly enriched in active enhancers (Fig. 4b). Highest increase (log2 fold change >2) was detected in PE and PP stages for FOXA2, PDX1 and GATA6. To check if these co-binding events were reflected in motif positioning, we performed a MEME-ChIP analysis. This analysis detected a de novo ONECUT1 motif (89% of peaks) central to peaks and a FOXA2 motif (24% of peaks), which preferentially binds 9–10 bps from the peak center (Fig. 4c). This also supports a physical proximity and possible interaction of ONECUT1 and FOXA2. These results support altogether that ONECUT1 is associated to activation of PE and PP specific enhancers by presumably  co-binding PDX1, GATA6 and FOXA2.

The previous analysis indicated that ONECUT1 is associated with novel active enhancers in PE and PP stages. We therefore investigated genomic loci of genes with PE and PP specific expression (Fig. 1c, e), which gained an active enhancer from GT to PE or PE to PP stages. The ONECUT1 gene has a large PP specific de novo active enhancer up-stream of its promoter, which was co-bound by ONECUT1, FOXA2, GATA6, and PDX1 (Fig. 4d). FOXA2 and NGN3 (NEUROG3) were two genes with high expression in PE/PP stages (Fig. 1c). Both had ONECUT1 binding sites associated with novel acquisition of an active enhancer at the PE stage by either co-binding with GATA6 or PDX1 (Fig. 4d). PROX1, GP2, and SOX9 were genes with high expression in PP stage (Fig. 1c, e). These genes had several de novo active enhancers mostly gained at PP stage (Fig. 4d). Notably, ONECUT1 co-binds with GATA6 and FOXA2 in most of these enhancers. In all enhancers, we observed the deposition of H3K4me1 marks and ATAC-seq starting from GT stages and preceding the activating marks H3K27ac. This supports the priming mechanism of ONECUT1 observed in Fig. 2d and provides examples of important pancreas genes being regulated by ONECUT1.

### ONECUT1 regulates islet cell genes

**Fig. 5:**
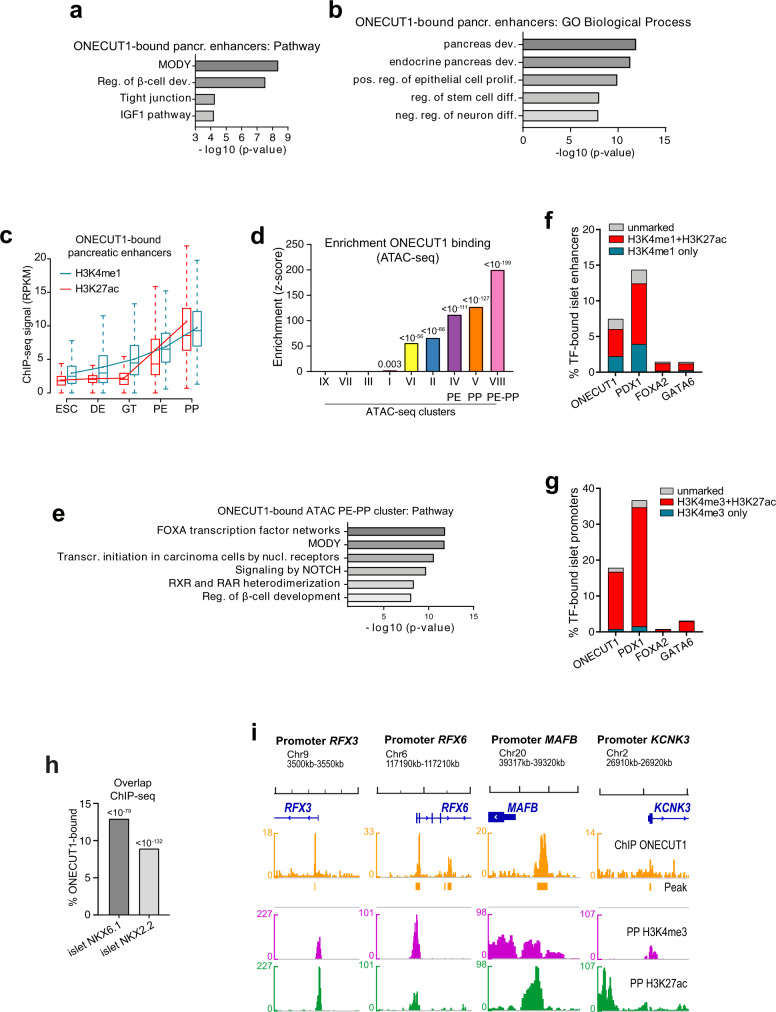
Pancreatic and islet enhancers are bound by ONECUT1. **a**, **b** Enrichment analysis (GREAT^36^) of ONECUT1-bound pancreatic enhancers (FG/PE and PE clusters from ref. ^29^) for “pathway terms” (**a**) from the MSigDB pathway genes subset CP and for “GO Biological Process” (**b**) from the Gene Ontology Consortium (Binomial test; one side). **c** Distribution of stage-specific H3K4me1 and H3K27ac ChIP-seq signal at ONECUT1-bound pancreatic enhancers (FG/PE and PE clusters from ref. ^29^). Boxplots depict median (center), interquartile range (box) and extreme values (whiskers), point and connecting line mean value. **d** Binding enrichment (*z*-score) test of ONECUT1 (ChIP-seq, PP stage) in distinct OC clusters of HUES8 ATAC-seq (*z*-test; one side). **e** Enrichment analysis (GREAT^36^) of ONECUT1-bound genes overlapping with PE/PP ATAC-seq (cluster VIII) for “pathway terms” (Binomial test; one side). **f**, **g** Overlap between ChIP-seq peaks of ONECUT1 (PP stage) and depicted TFs with islet enhancers (**f**) and islet promoters (**g**) together with distinct histone modification as defined by ref. ^45^. **h** Overlap between ONECUT1 ChIP-seq peaks (PP stage) with islet TF peaks (NKX6.1 and NKX2.2 ChIP-seq from human islets^45^; intersection test, one side^84^). **i** Interactive Genome Viewer (IGV) plots illustrates ONECUT1 binding peaks and histone marks in promoter regions for selected endocrine genes. Genomic locations refer to human genome assembly GRCh37 (Hg19).

Subsequent GREAT analysis highlighted the role of ONECUT1 in specification toward a β-cell-specific program with top-ranked terms like “MODY” or “regulation of β-cell development” (Fig. 5a, b). ONECUT1-bound pancreatic enhancers accumulated H3K4me1 around the GT stage, while H3K27ac accumulated only from GT stage on, surpassing H3K4me1 levels at the PP stage (Fig. 5c). This is in line with the observation that pancreatic enhancers acquired a poised state around the GT stage prior to activation^29^ and indicates that ONECUT1 might be crucial for priming and activating enhancers formed at the PE stage.

To correlate ONECUT1 binding with accessible chromatin, we mapped ONECUT1 ChIP-seq binding peaks from the PP stage to OC clusters from ATAC-seq experiments. ONECUT1 binding was most evident in the transition cluster VIII (PE-PP), followed by cluster V (PP), which indicates that ONECUT1 binding might start at the PE stage to promote a PP program (Fig. 5d). GREAT analysis of the PE-PP cluster identified “MODY” and “regulation of β-cell development” top-ranked together with “FOXA transcription” and “RXR/RAR heterodimerization” (Fig. 5e). Of note, it has been reported that retinoic acid signaling is crucial for establishing the PP stage^44^. These results indicate that ONECUT1 is bound to active enhancers starting to be accessible at the PE and/or PP stages, supporting its central regulatory function within the pancreatic transcriptional network.

Moreover, ONECUT1 bound to active enhancers (7%) and promoters (18%) (Fig. 5f, g) and to regions bound by NKX6.1 (13%) and NKX2.2 (9%) in human islets^45^ (Fig. 5h). Interestingly, a large proportion of the few PP ONECUT1 promoter-bound sites were proximal to genes expressed in islets (Fig. 5g), which included promoters of key genes such as *RFX3, RFX6, KCNK3*, and *MAFB* active in β-cells (Fig. 5i). In summary, *ONECUT1* expression is sustained from the PE to the PP stage and the protein binds to active pancreas-specific enhancers as well as active regulatory regions of islet cells.

## Discussion

Here, we comprehensively characterized transcriptional and chromatin changes in pancreatic in vitro differentiation. Our global resource consists of transcriptional (RNA-seq), chromatin (ATAC-seq) and regulatory (ONECUT1 ChIP-seq) analyses at distinct pancreas differentiation stages. These experiments were complemented with our own and publicly available ChIP-seq data measuring histone marks (H3K4me3, H3K4me1, H3K27ac) and TF (FOXA2, GATA6, NKX6.1, and PDX1) binding at the PP stage. Computational analysis of transcriptional and OC data in our human stem cell differentiation approach revealed expression of ONECUT1 during early pancreatic development at PE and PP stage together with key pancreatic TFs consistent with data from mouse studies^32,33,46^. Our protocol for differentiating HUES8 cells towards the pancreatic lineage indicated robust induction of the endocrine gene expression program at PE and PP stage. This suggests that our protocol is suitable to explore early transcriptional regulators as well as the impact of so far unknown gene variants impairing endocrine development in diseases such as diabetes. Other cell lines revealed similar but slightly different patterns. Depending on genes to be explored and e.g., the availability of certain reporter cell lines, it might be beneficial to follow gene expression in different protocols and cell lines.

Moreover, ONECUT1-bound genes were enriched for pancreatic and endocrine development. This is in line with Onecut1 knock-out mouse data, demonstrating that Onecut1 regulates Pdx-1 and Ngn3, which are important for pancreatic endocrine specification^32,33,46^. Onecut1 and Pdx1 interact during murine endocrine development and the combined function of both transcription factors is necessary for β-cell maturation and adaptation^46–48^. This correlates with observed protein interaction^16^ as well as our motif analysis revealing co-binding to active enhancers during human pancreas differentiation. Our chromatin-regulatory and cis-regulatory binding maps provide evidence that gene transcription regulated by ONECUT1 is located in clusters of OC, which are co-bound within enhancer regions by a complete suite of physically interacting TFs normally enriched in islets^45,49,50^. Our analysis further suggests combined priming activity of ONECUT1 with TFs FOXA2, GATA6, PDX1, and NKX6.1 by preparing and activating enhancers of important pancreatic regulators for expression. Previously, the major pancreas TF PDX1 was found to regulate β-cell development by binding mainly intron and intergenic but also promoter regions of pancreatic genes such as RFX6, HNF1B, and MEIS1^51^. Our analysis further suggests a role of ONECUT1 priming activity for preparing loci of important pancreatic regulators for expression, e.g., NGN3 (NEUROG3) important for generation of functional β-cells^52^. Similarly, SOX9, important for regulating NGN3 as well as other TFs during endocrine development^53^, and PROX1, playing a role in pancreatic progenitor specification and proliferation as well as insulin secretion^26,54^, are regulated by this TF network and therefore contribute to timed development. This is in line with data gathered in our recent study indicating that binding of ONECUT1 at enhancers triggers an active state in PPs to allow expression of islet-specific TFs NKX6.1, NKX6.2, and NKX2.2^16^. Similar binding to regulatory regions of genes important for endocrine cell development was observed for Nkx6.1 in mice^55,56^. This may suggest that ONECUT1 plays a similar role inducing the pancreatic transcriptional program and priming cells for further endocrine differentiation.

Binding of ONECUT1 to islet enhancers included regions of genes encoding proteins such as INSM1, important for maturation of endocrine precursor cells and maintenance of adult pancreatic β‐cells^57,58^ and FOXA2, a regulator for pancreas development and adult pancreatic β‐cell function^59,60^. In addition, we also observed ONECUT1 binding to active promoters of genes important for β-cell development and function. RFX6 coordinates islet development and β-cell identity in human and mouse but also controls the number of PPs^52,61,62^. Similarly, we found promoter binding to *RFX3*, important for differentiation and function of β-cells in mice^63^. We also found ONECUT1 binding to the promoter of the highly expressed potassium channel *KCNK3* (also known as *TASK-1*) involved in modulating insulin secretion and glucose homeostasis^64^, as well as in the promoter of *MAFB*, which is required for regulating development of α- and β-cells in human and in mice^65,66^. Altogether, our global analyses establish ONECUT1 within the interconnected and tightly controlled regulatory network of pancreatic TFs during pancreatic and endocrine development. Although our data suggest a role for ONECUT1 in pancreatic and endocrine development, limitations apply to bulk cell analysis of differentiation stages due to the complex heterogeneity and interconnectedness of cell populations. Bulk sequencing data illustrate an average characteristic of cell populations and are not able to resolve the complex composition and multiple pathways at a specific differentiation stage. In order to address a possible bias, further in-depth analysis of single-cell data will be needed.

We recently reported *ONECUT1* as a gene involved in various forms of human diabetes, ranging from severe neonatal syndromic diabetes (biallelic mutations) and young-onset non-autoimmune diabetes with incomplete penetrance (monoallelic mutations) to common T2D (regulatory variants)^16^. Further analysis in our human stem cell differentiation model provides evidence that pathogenic ONECUT1 variants disturb the development of pancreatic progenitors and β-like cells due to their diminished capability to activate factors such as *NKX6.1, NKX6.2*, and *NKX2.2* important for β-cell formation.

The present study provides further understanding on the role of ONECUT1 within a tightly controlled network of genes involved in pancreatic and endocrine development. Remarkably, several of the genes belonging to this network are also involved in monogenic and in multifactorial diabetes^67,68^ further supporting the relevance of our human stem cell differentiation model to help identify and study genes and variants involved in diabetes.

## Methods

### Stem cell culture

Permission for culture and pancreatic differentiation of human embryonic stem cell (hESC) lines was obtained from the Robert Koch Institute within the “79. Genehmigung nach dem Stammzellgesetz, AZ 3.04.02/0084”. HUES8 cell line was received from Harvard University and MEL1 from Stem Cells Ltd. Cells were cultured on plates coated with hESC Matrigel in mTESR1 (STEMCELL Technologies) medium at 5% CO_2_, 5% O_2_, and 37 °C and change of medium was performed daily. For splitting our feeder-free single cell cultures twice a week, cells washed with PBS were dissociated for 3–5 min at 37 °C with TrypLE Express (Invitrogen) followed by diluting with blank medium to stop enzyme reaction. Cell suspension was centrifuged at 800 rpm for 5 min and cell pellet was resuspended in mTESR1 medium supplemented with 10 µM ROCK inhibitor (Abcam). Mel1 INS^GFP/W^ cells were cultured according to Nair et al.^17^.

### Differentiation of PSCs into pancreatic progenitor cells

Pancreatic differentiation is based on published protocols^69,70^. Basal media BE1: MCDB131 (Invitrogen) with 0.8 g/l glucose (Sigma), 1.174 g/l sodium bicarbonate (Sigma), 0.5% fatty acid free BSA (Proliant), 2 mM l-Glutamine; BE3: MCDB131 with 3.32 g/l glucose, 1.754 g/l sodium bicarbonate, 2% FAF-BSA, 2 mM l-Glutamine, 44 mg/l l-Ascorbic acid, 0.5% ITS-X.

For differentiation, culture plates were coated with growth factor reduced Matrigel (BD, 354230) and 300,000 hESCs per 24-well were seeded in mTESR1 containing 10 µM ROCK inhibitor. The following day, differentiation was initiated when cells reached 80% confluence and culture was transferred in a 5% CO_2_ incubator at 37 °C with daily medium change. After washing with PBS (Sigma), cells received for 24 h BE1 medium with 2 µM CHIR99021 (Axon MedChem) and 100 ng/ml Activin A (R&D). The following 2 days, cells were cultured in BE1 supplemented with 100 ng/ml Activin A and 5 ng/ml bFGF (R&D). At DE stage, cells were maintained for three days in BE1 with 50 ng/ml FGF10 (R&D), 0.75 µM Dorsomorphin (Sigma) and 3 ng/ml Wnt3a (Peprotech) in BE1 followed by BE3 with 0.25 µM SANT-1 (Sigma), 200 nM LDN-193189 (Sigma), 2 µM Retinoic acid (Sigma), and 50 ng/ml FGF10 for three days. From day 9 to 13, cells were maintained in BE3 containing 100 ng/ml EGF (R&D), 200 nM LDN, 330 nM Indolactam V (Stem Cell Technologies) and 10 mM Nicotinamide (Sigma). MEL1 INS^GFP/W^ were differentiated as described in the protocol published by Nair et al.^17^.

### Immunofluorescence staining

Human ESCs differentiated on µ-Plate 24-wells (Ibidi) were used for in-well immunofluorescence staining. Cells were washed with PBS followed by fixation in 4% PFA solution for 20 min at RT. Subsequently, fixed cells were washed three times with PBS and background staining was quenched with 50 mM NH_4_Cl for 10 min. After washing with PBS, cells were incubated in PBS containing 0.1% Triton-X and 5% normal donkey serum (blocking) at room temperature for 45 min and subsequently, in blocking solution with primary antibodies overnight at 4 °C. Cells were washed twice with PBS containing 0.1% Triton-X and 2% normal donkey serum (wash solution) followed by incubation in blocking solution containing secondary antibodies at room temperature for 1.5 h. Finally, cells were washed with PBS and nuclei were stained with 500 ng/ml DAPI. Images were acquired on a Keyence Biozero BZ-9000 microscope. Antibodies used: OCT3/4 (Santa Cruz; sc-5279; 1:200), NANOG (Cell Signaling; #3580; 1:100), SOX17 (R&D; AF1924; 1:500), PDX1 (R&D; AF2419; 1:500), NKX6.1 (DSHB; F55A10 concentrate; 1:100) in combination with Alexa-conjugated secondary antibodies from Invitrogen.

### Immunofluorescence staining on paraffin tissue sections

Spheres derived from MEL1 were fixed for 15 min at RT with 4% paraformaldehyde, washed in PBS and subsequently embedded in 2% agarose (Sigma). Samples were dehydrated and paraffin embedded, followed by sectioning at 5 μm thickness. For staining, rehydrated sections were treated with antigen retrieval solution (Biogenex), blocked (CAS-Block, Life Technologies with 0.2% Triton-X 100, Sigma) and incubated in primary antibodies at 4 °C overnight. After washing in PBS with 0.1% Tween20, sections were incubated with secondary antibodies for 45 min at room temperature. Finally, slides were washed in PBS-T and PBS and mounted with Vectashield. Images were acquired using a Zeiss ApoTome. The following antibodies were used: SOX17 (R&D; AF1924; 1:500), PDX1 (R&D; AF2419; 1:500), NKX6.1 (DSHB; F55A12 concentrate; 1:150), and Alexa-conjugated secondary antibodies from Invitrogen. Nuclei were visualized with DAPI.

### Flow cytometry

Markers c-Kit (CD117; APC conjugated; Invitrogen; CD11705; 1:100), CXCR4 (CD184; PE conjugated; Life Technologies; MHCXCR404; 1:33), SOX17 (Alexa488 conjugated; BD Biosciences; 562205; 1:100) and FOXA2 (PE conjugated; BD Biosciences; 561589; 1:100) were quantified for definitive endoderm (DE), PDX1 (PE conjugated; BD; 562161; 1:35) and NKX6.1 (Alexa647 conjugated; BD; 563338; 1:35) were analyzed for pancreatic endoderm (PE) and pancreatic progenitor (PP) using BD LSC II flow cytometer or BD FACSAria II cell sorter with FACSDiva software version 8.0.1 (BD Biosciences) and FlowJo 10.5.0 as described by Philippi et al.^16^.

### RNA sequencing

Total RNA was isolated from different stages of pancreatic cells using GeneJET RNA Purification Kit (Thermo Fisher Scientific) following the manufacturer’s protocol. After quality check, up to 1 µg of total RNA with RNA integrity values (RIN) > 8 was used for poly-A enrichment followed by library preparation using the TrueSeq stranded mRNA Kit from Illumina (HUES8 *n* = 6, Mel1 *n* = 4). For HUES8, a HiSeq 3000 system (Illumina, single read, 1 × 50 bp) at the Biomedical Sequencing Facility (BSF) of the CeMM in Vienna, Austria, was used, whereas for Mel1 RNA samples a HiSeq 2500 system (Illumina, single read, 1 × 50 bp) was utilized for RNA sequencing at the Leibnitz Institute in Jena, Germany.

We used STAR aligner (Version 2.5.2b;^71^) to align reads on human genome hg38 with parameters as in ENCODE project. Ensembl Transcriptome Annotation e87 was used as transcriptome reference. Normalization and differential expression (DE) was performed with DESeq2 (version 1.22.1)^72^. We only considered further genes with more than 10 reads, an adjusted *p*-value < 0.05 and abs(FC) > 1. We next performed clustering of DE genes with a fuzzy c-means algorithm (R package e1071). The same approach was used for HUES8 (ESC, DE, PE, and PP stages on WT), MEL1 (ES, DE, PE, and PP), and CyT49 cells (publicly available at E-MTAB-1086^30^).

We performed functional analysis with gene set enrichment analysis (GSEA version 3.0^73^) to analyze genes. For this, we used the log2-fold change (FC) and the GSEAPreranked function with 1000 permutations in default settings. We manually derived genes sets from Cebola et al.^22^, which contains 500 pancreatic multipotent progenitor cell (MPC)-specific genes, and from Hrvatin et al.^74^ for endocrine development genes. We used ToppFun for Gene Ontology enrichment (FDR correction, adjusted *p*-value cut off at 0.5)^75^.

### ATAC sequencing

For ATAC sequencing, differentiated pancreatic cells were harvested and 50,000 cells were directly lysed in tagmentation buffer containing TDE1 Tagment DNA enzyme, Digitonin and protease inhibitor cocktail (Nextera DNA Library Preparation Kit, Illumina). Lysates were incubated for 30 min at 37 °C and subsequently purified with the MicroElute Kit (Qiagen) according to the manufacturer’s protocol. Samples (*n* = 3) were stored at −20 °C until further processing and analysis at BSF in Vienna.

### ChIP sequencing

The ChIP-IT High-Sensitivity kit (Active Motif) was utilized following the manufacturer’s protocol. Briefly, aggregates of pancreatic cells containing approximately 10^7^ cells fixed for 15 min in an 11.1% formaldehyde solution were applied to chromatin extraction. Cells were lysed in a Dounce homogenizer and DNA was sheared with a Bioruptor®Plus (Diagenode) sonicator, on high for 3 × 5 min (30 s on, 30 s off). For subsequent immunoprecipitation, 10–30 μg of sheared chromatin was mixed with 4 μg primary antibody (HNF6 H-100, sc-13050, Santa Cruz; or NKX6.1 RES310, AB2024, Beta Cell Biology Consortium) and incubated over night at 4 °C on an end-to-end rotator. The chromatin-antibody solution was incubated with Protein G agarose beads for 3 h at 4 °C on the rotator and subsequently samples were processed according to the ChIP-IT High-Sensitivity instructions with an incubation at 65 °C for 2 h to reverse crosslinks and purify DNA. KAPA DNA Library Preparation Kits for Illumina® (Kapa Biosystems) were used to construct DNA libraries. For library sequencing, a HiSeq 4000 System (Illumina®) with single-end reads of 50 bp in the Institute for Genomic Medicine (IGM) core research facility at the University of California at San Diego (UCSD) was used. Additional details on the employed data sets are reported also in refs. ^35,59^

### Bioinformatics analysis of ATAC-seq and ChIP-seq

First, we trimmed reads with skewer (Version 0.2.1;^76^) and next used Bowtie2 (Version 2.3.4.2;^77^) to align the human hg19 genome. We only considered properly mapped read pairs in ATAC-seq data. Also, we removed duplicated reads. Peak calling was performed with MACS2 (2.1.2;^78^) with FDR of 5%. This allowed us to find stage-specific peaks by using corresponding input DNA and replicates. For the ChIP-seq data of TFs (ONECUT1, FOXA1, FOXA2, PDX1, and NKX6.1^29,35^), we also detected de novo motifs with MEME-ChIP (Version 4.12;^79^). The previous methods were also applied to publicly available histone data (H3K4me1, H3K27ac, GSE54471^29^ and H3K4me3, ArrayExpress E-MTAB-1086^30^).

Only for ATAC-seq data, we also used a differential peak calling approach (THOR—Version 0.11.6;^80^) to detect differential peaks between consecutive steps (ESC vs. DE, DE vs. GT, GT vs. PE, and PE vs. PP). We only considered peaks with *p*-value < 10^−5^. Next, we built a peak vs. condition count matrix by merging all peaks with bedtools^81^ and quantification of the number of reads per peak. This matrix was first normalized by library size followed by a *z*-transformation (removal of mean and scaling to unit variance). Next, we grouped the peaks by clustering with fuzzy c-medoids. Pearson correlation was used as similarity metric.

We performed a footprinting analysis to dissect the regulatory program driving distinct differentiation stages. For this, we use HINT-ATAC (Version 0.11.8;^14^) to detect footprints at each condition (ESC, DE, GT, PE, and PP). Next, we characterized TFs associated to the footprints with motif matching with Regulatory Genomics Toolbox (RGT; www.regulatory-genomics.org/rgt). JASPAR 2018^82^ was used as a TF motif database. Next, we performed differential footprinting analysis comparing subsequent differentiation steps (ESC vs. DE, DE vs. GT, GT vs. PE, and PE vs. PP) and only considering TFs with significant changes (p-value < 0.05).

Histone and ATAC-seq heatmaps were performed with Deeptools^83^. We used RGT (RGT; www.regulatory-genomics.org/rgt) to perform a binding enrichment test comparing gene sets with peaks. This is based on a *z*-score test, which tests if the number of genes close to a peak (tss +/− 20 kb) is higher than in random peaks (1000 randomizations). An intersection test^84^ was used to assess the overlap between two peak sets. *p*-values were adjusted for multiple test correction using the Bonferroni method.

### Statistics and reproducibility

For RNA-seq and ATAC-seq, at least three independent experiments were performed with the number of biological replicates mentioned in figure legend. Statistical analyses were performed in R (version 4.0.2) or by the software described for each analysis test. The statistical method used for each individual analysis are described in the corresponding figure. All reported *p*-values based on multi-comparison tests were corrected using the Bonferroni method.

### Reporting summary

Further information on research design is available in the Nature Research Reporting Summary linked to this article.

## Supplementary information

Supplementary Information

Supplementary Data 1

Description of Additional Supplementary Files

Reporting Summary

## Data Availability

All own sequencing data (RNA-seq, ATAC-seq, and ChIP-seq) and pre-processed files (count matrices, peaks, and genomic profiles) are deposited at Gene Expression Omnibus (GSE167606). We also provide all genomic tracks and genomic regions (peaks) from both own and public data in Zenodo (10.5281/zenodo.4559829). Public available data include histome ChIP-seq (H3K4me1, H3K27ac, GSE54471^29^, and H3K4me3, E-MTAB-1086^30^) and transcription factor ChIP-seq (FOXA1, FOXA2; GSE54471^29^; and PDX1, GSE149148^35^). Source data associated to figure panels are provided in the Supplementary Data 1.
