# Supplementary material for: Transcriptional changes and the role of ONECUT1 in hPSC pancreatic differentiation
Source: Commun Biol. 2021 Nov 17;4:1298. doi: 10.1038/s42003-021-02818-3 (PMC8599846; doi:10.1038/s42003-021-02818-3)
Supplement: Supplementary file 1 — Supplementary Information [file 42003_2021_2818_MOESM1_ESM.pdf]

Supplementary Figure 1

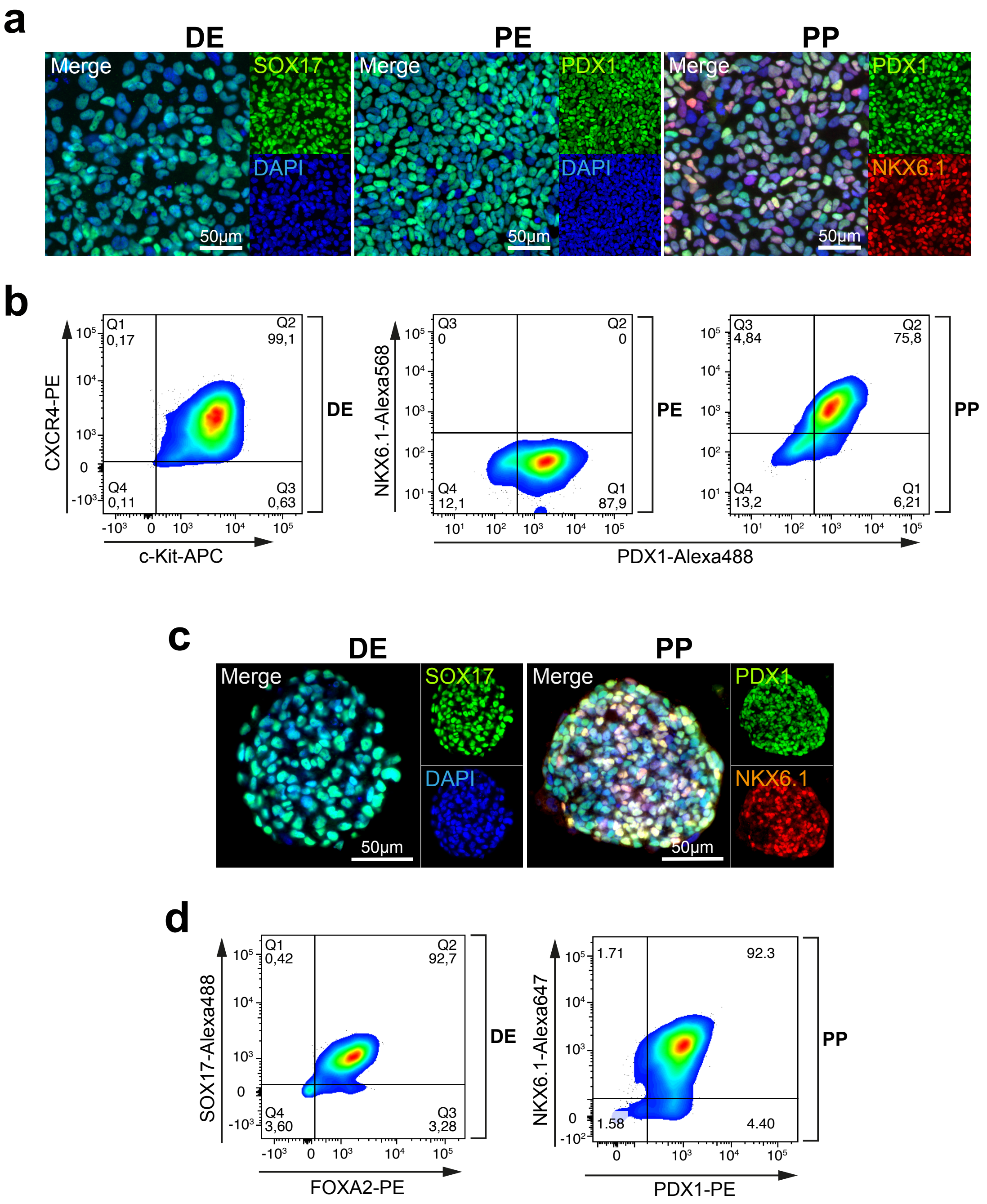

Pancreatic differentiation of different cell lines. (a) Differentiation efficiency at DE, PE, and PP stages in HUES8 WT cells. Representative images show immunofluorescence stainings for stage-specific markers. **(b)** Marker expression during differentiation was quantified by flow cytometry using CXCR4 and c-Kit as DE markers and PDX1 and NKX6.1 as PE/PP markers. **(c,d)** In MEL1 INS-GFP cells, differentiation efficiency was assessed at DE and PP cell stages. Representative immunofluorescent stainings of respective markers are shown (c). SOX17 and FOXA2 as DE markers and PDX1 and NKX6.1 as PE/PP markers were quantified by flow cytometry as illustrated by representative plots (d).
