# Supplementary material for: Transcriptional changes and the role of ONECUT1 in hPSC pancreatic differentiation
Source: Commun Biol. 2021 Nov 17;4:1298. doi: 10.1038/s42003-021-02818-3 (PMC8599846; doi:10.1038/s42003-021-02818-3)
Supplement: Supplementary file 4 — Reporting Summary [file 42003_2021_2818_MOESM4_ESM.pdf]

nature portfolio

Corresponding author(s): Ivan G. Costa, Alexander Kleger, Cécile Julier

Last updated by author(s): Oct 7, 2021

Reporting Summary

Nature Portfolio wishes to improve the reproducibility of the work that we publish. This form provides structure for consistency and transparency in reporting. For further information on Nature Portfolio policies, see our Editorial Policies and the Editorial Policy Checklist.

Statistics

For all statistical analyses, confirm that the following items are present in the figure legend, table legend, main text, or Methods section.

n/a	Confirmed	
☐	☒	The exact sample size (*n*) for each experimental group/condition, given as a discrete number and unit of measurement
☐	☒	A statement on whether measurements were taken from distinct samples or whether the same sample was measured repeatedly
☐	☒	The statistical test(s) used AND whether they are one- or two-sided<br>*Only common tests should be described solely by name; describe more complex techniques in the Methods section.*
☐	☒	A description of all covariates tested
☐	☒	A description of any assumptions or corrections, such as tests of normality and adjustment for multiple comparisons
☐	☒	A full description of the statistical parameters including central tendency (e.g. means) or other basic estimates (e.g. regression coefficient) AND variation (e.g. standard deviation) or associated estimates of uncertainty (e.g. confidence intervals)
☐	☒	For null hypothesis testing, the test statistic (e.g. *F*, *t*, *r*) with confidence intervals, effect sizes, degrees of freedom and *P* value noted<br>*Give P values as exact values whenever suitable.*
☒	☐	For Bayesian analysis, information on the choice of priors and Markov chain Monte Carlo settings
☒	☐	For hierarchical and complex designs, identification of the appropriate level for tests and full reporting of outcomes
☒	☐	Estimates of effect sizes (e.g. Cohen's *d*, Pearson's *r*), indicating how they were calculated

Our web collection on statistics for biologists contains articles on many of the points above.

Software and code

Policy information about availability of computer code

Data collection	Sequencing data was generated from own cells samples or in some cases retrieved from open sources as stated in methods section. No software was used for data collection.
Data analysis	Sequencing data analysis used the read trimmer skewer (Version 0.2.1; 24), the aligner Bowtie2 Version 2.3.4.2; 25, the peak caller MACS2 (2.1.2; 26); the de-novo motif tool MEME-ChIP (Version 4.12; 27), the differential peak caller THOR (Version 0.11.6; 28) , the footprinting methods HINT-ATAC (Version 0.11.8; 29); the TF motif database JASPAR (version 20180; the MotifMatching from Regulatory Genomics Toolbox (www.regulatory-genomics.org/rgt; version 0.12.3); the enrichment analaysis GREAT (version 3), GSEA (version 3) and ToppGene Suite (version 35) and the ChIP-seq vizualisation tool Deeptools (version 3.3.00).

For manuscripts utilizing custom algorithms or software that are central to the research but not yet described in published literature, software must be made available to editors and reviewers. We strongly encourage code deposition in a community repository (e.g. GitHub). See the Nature Portfolio guidelines for submitting code & software for further information.

Data

Policy information about availability of data

All manuscripts must include a data availability statement. This statement should provide the following information, where applicable:

- Accession codes, unique identifiers, or web links for publicly available datasets
- A description of any restrictions on data availability
- For clinical datasets or third party data, please ensure that the statement adheres to our policy

All own sequencing data (RNA-seq, ATAC-seq and ChIP-seq) and pre-processed files (count matrices, peaks and genomic profiles) are deposited at Gene Expression Omnibus (GSE167606). We also provide all genomic tracks and genomic regions (peaks) from both own and public data in Zenodo (10.5281/zenodo.4559829).

Public available data include histome ChIP-seq (H3K4me1, H3K27ac, GSE5447128 and H3K4me3, E-MTAB-108629) and transcription factor ChIP-seq (FOXA1, FOXA2; GSE5447128; and PDX1, GSE14914835). Source data associated to figure panels are provided in the Supplementary Data File.

Field-specific reporting

Please select the one below that is the best fit for your research. If you are not sure, read the appropriate sections before making your selection.

- [x] Life sciences
- [ ] Behavioural & social sciences
- [ ] Ecological, evolutionary & environmental sciences

For a reference copy of the document with all sections, see nature.com/documents/nr-reporting-summary-flat.pdf

Life sciences study design

All studies must disclose on these points even when the disclosure is negative.

Sample size	No statistical methods were used to calculate sample size for our biological experiments. At least three independent experiments were performed with the number of biological replicates mentioned in the manuscript. Sample size was selected based on previous studies in the field.
Data exclusions	We did not exclude data from analysis.
Replication	In general, results of biological experiments were determined from at least three independent experiments with numbers stated in methods section and figure legend.
Randomization	Randomization was not relevant for our study.
Blinding	Data analyses were not blinded, since genetic analyses require the clinical status of subjects.

Reporting for specific materials, systems and methods

We require information from authors about some types of materials, experimental systems and methods used in many studies. Here, indicate whether each material, system or method listed is relevant to your study. If you are not sure if a list item applies to your research, read the appropriate section before selecting a response.

Materials & experimental systems

n/a	Involved in the study
[ ]	[x] Antibodies
[ ]	[x] Eukaryotic cell lines
[x]	[ ] Palaeontology and archaeology
[x]	[ ] Animals and other organisms
[x]	[ ] Human research participants
[x]	[ ] Clinical data
[x]	[ ] Dual use research of concern

Methods

n/a	Involved in the study
[ ]	[x] ChIP-seq
[ ]	[x] Flow cytometry
[x]	[ ] MRI-based neuroimaging

Antibodies

Antibodies used

Primary Antibodies; Host; Company; Catalogue Number; Dilution; Application
HNF6 (H100); rabbit; Santa Cruz; sc-13050; -; ChIP
c-KIT-APC conjugated; mouse; Invitrogen; CD11705; 1:100; FC
CXCR4-PE conjugated; mouse; Life Technologies; MHCXCR404; 1:33; FC
NKX6.1-647 conjugated; mouse; BD; 563338; 1:35; FC
PDX1-PE conjugated; mouse; BD; 562161; 1:35; FC
NKX6.1; mouse; DSHB; F55A12 concentrate; 1:150; FC, IF
NKX6.1; mouse; DSHB; F55A10 concentrate; 1:100; IF
PDX1; goat; R&D; AF2419; 1:500; FC, IF
NANOG; rabbit; Cell Signaling; #3580; 1:100; IF
Oct3/4; mouse; Santa Cruz; sc-5279; 1:200; IF
SOX17; goat; R&D; AF1924; 1:500; IF
SOX17-Alexa488 conjugated; mouse; BD Biosciences; 562205; 1:100; FC
FOXA2-PE conjugated; mouse; BD Biosciences; 561589; 1:100; FC

Validation

Primary antibodies used for this study were recommended for the applications by the manufacturer and/or used in previous publications. Relevant articles are:

Weedon, Michael N., et al. "Recessive mutations in a distal PTF1A enhancer cause isolated pancreatic agenesis." Nature genetics 46.1 (2014): 61.

Alpern, Daniil, et al. "TAF4, a subunit of transcription factor II D, directs promoter occupancy of nuclear receptor HNF4A during post-natal hepatocyte differentiation." Elife 3 (2014): e03613.

Ballester, Benoit, et al. "Multi-species, multi-transcription factor binding highlights conserved control of tissue-specific biological pathways." Elife 3 (2014): e02626.

Korytnikov, Roman, and Maria Cristina Nostro. "Generation of polyhormonal and multipotent pancreatic progenitor lineages from human pluripotent stem cells." Methods 101 (2016): 56-64.

Kelly, Olivia G., et al. "Cell-surface markers for the isolation of pancreatic cell types derived from human embryonic stem cells." Nature biotechnology 29.8 (2011): 750.

Pedersen, Inger L., et al. "Generation and characterization of monoclonal antibodies against the transcription factor Nkx6. 1." Journal of Histochemistry & Cytochemistry 54.5 (2006): 567-574.

Rosado-Olivieri, Edwin A., et al. "YAP inhibition enhances the differentiation of functional stem cell-derived insulin-producing β cells." Nature communications 10.1 (2019): 1464.

Zhou, Ting, et al. "A hPSC-based platform to discover gene-environment interactions that impact human β-cell and dopamine neuron survival." Nature communications 9.1 (2018): 4815.

Teo, Adrian KK, et al. "Activin and BMP4 synergistically promote formation of definitive endoderm in human embryonic stem cells." Stem Cells 30.4 (2012): 631-642.

Eukaryotic cell lines

Policy information about cell lines

Cell line source(s)	HUES8 cell line was received from Harvard University, MEL-1 from Stem Cells Ltd, and CyT49 cell line from ViaCyte, Inc. Stem Cell Derivation.
Authentication	For HUES8, a DNA profile generated at 8 STR regions using nonaplex PCR was compared to the parental profile (reference databases) and confirmed the authenticity of the cell line. Pluripotency of human embryonic stem cells and human induced pluripotent stem cells was tested through the expression of pluripotency-associated markers OCT3/4 and NANOG.
Mycoplasma contamination	All cell lines are regularly tested for mycoplasma contamination using the MycoProbe Mycoplasma Detection Kit (R&D). For cell lines used within this manuscript, test results were negative for contamination.
Commonly misidentified lines (See ICLAC register)	No commonly missidentified cell lines were used.

ChIP-seq

Data deposition

☒ Confirm that both raw and final processed data have been deposited in a public database such as GEO.

☒ Confirm that you have deposited or provided access to graph files (e.g. BED files) for the called peaks.

Data access links<br>*May remain private before publication.*	ChIP-seq generated for this work has been deposited at Gene Expression Omnibus (GSE167606).
Files in database submission	GEO data includes reads (fastq), signals (bw) and peaks (bed) for all replicates and stages.
Genome browser session (e.g. UCSC)	*Provide a link to an anonymized genome browser session for "Initial submission" and "Revised version" documents only, to enable peer review. Write "no longer applicable" for "Final submission" documents.*

Methodology

Replicates	ChIP-seq were performed as single experiments. Input DNA of matching cells was used as control.
Sequencing depth	Experiments of GT and PP HNF6 obtained respectively 25.055.805 and 22.902.567 mapped single end reads (50 bps). Input files (GT and PP) had respectively 22.354.866 and 24.139.204 mapped reads.
Antibodies	HNF6 (H100), rabbit, Santa Cruz, sc-13050; NKX6.1 RES310, AB2024, Beta Cell Biology Consortium
Peak calling parameters	We used MACS2 (2.1.2; 26) with FDR of 5% to find condition-specific peaks using corresponding input DNA.
Data quality	We find 255 GT stage HNF6 peaks, 66373 PP stage HNF6 peaks with a FDR of 5%.

Software	Reads were trimmed with skewer (Version 0.2.1; 24) and aligned with Bowtie2 to the human hg19 genome (Version 2.3.4.2; 25). We used MACS2 (2.1.2; 26) with FDR of 5% to find condition-specific peaks using corresponding input DNA. We have performed de-novo analysis with MEME-ChIP (Version 4.12; 27).

Flow Cytometry

Plots

Confirm that:

- [x] The axis labels state the marker and fluorochrome used (e.g. CD4-FITC).
- [x] The axis scales are clearly visible. Include numbers along axes only for bottom left plot of group (a 'group' is an analysis of identical markers).
- [x] All plots are contour plots with outliers or pseudocolor plots.
- [x] A numerical value for number of cells or percentage (with statistics) is provided.

Methodology

Sample preparation	Human ESC grown and differentiated in monolayer were harvested with TrypLE Express (Invitrogen). For surface marker analysis, cells were washed with FACS buffer (2% FCS in PBS) followed by blocking (10% FCS in FACS buffer). Finally, cell were incubated in FACS Buffer containing conjugated antibodies. Before analysis, cells were washed twice and costained with DAPI to assess cell viability.<br>For intracellular marker staining, cells were fixed with PFA solution (PBS with 4% PFA and 10% Sucrose). After fixation, cells were washed twice with PBS and blocked (5% donkey serum and 0.1% Triton-X-100 in PBS). Cells were incubated overnight at 4°C with primary antibodies in Blocking Solution. The next day after washing, cells were incubated with secondary antibodies conjugated with Alexa Fluor fluorochromes.<br>Before analysis, samples were filtered through a 50-µm mesh.
Instrument	BD LSC II flow cytometer, BD FACSAria II cell sorter
Software	FACSDiva software version 8.0.1 (BD Biosciencies) and FlowJo 10.5.0 was used for data analysis.
Cell population abundance	No cell sorting was performed within this manuscript.
Gating strategy	Forward and side scatter gating was used to remove debris while retaining cells based on size and or complexity. Moreover, doublets were removed by pulse geometry gating. For live-cell analysis, DAPI was added to exclude dead cells. Finally, different populations were gated according to samples negative for the respective marker.

- [ ] Tick this box to confirm that a figure exemplifying the gating strategy is provided in the Supplementary Information.
